# Start-up of a full-scale two-stage partial nitritation/anammox (PN/A) process treating reject water from high solid anaerobic sludge digestion (HSAD)

**DOI:** 10.1016/j.wroa.2024.100259

**Published:** 2024-09-23

**Authors:** Shuyan Zhou, Hui Gong, Enhui Xu, Xiang Chen, Xiankai Wang, Hang Wang, Danyang Zhu, Yanyan Zhang, Jing Yang, Guowei Gu, Xiaohu Dai

**Affiliations:** a College of Environmental Science and Engineering, State Key Laboratory of Pollution Control and Resources Reuse, Tongji University, Shanghai 200092, China; b YANGTZE Eco-Environment Engineering Research Center, China Three Gorges Corporation, Wuhan 430010, China; c National Engineering Research Center of Eco-environment Protection for Yangtze River Economic Belt, Wuhan 430010, China

**Keywords:** High solid anaerobic digestion, Two-stage, Partial nitritation/anammox, Full-scale, Start-up

## Abstract

High solid anaerobic digestion (HSAD) achieves the benefits of high volumetric loading rates and lower reject water production, which, however, results in much more concentrated reject water with a remarkable increase in organics and nitrogen compared with that from conventional AD with low solid content. The high concentrations of ammonium (2000–3500 mg/L) and COD (3000–4000 mg/L) were reported to exert inhibition on anammox bacteria (AnAOB), posing challenges to the application of the partial nitritation/anammox (PN/A). To date, no cases of PN/A process start-up for sludge HSAD reject water were reported. This study demonstrated the start-up process of a 480 m^3^/d PN/A project without anammox sludge inoculation and treating HSAD reject water from a centralized dewatered sludge treatment plant. The project did not construct new infrastructures but utilized previously constructed tanks to upgrade the process from existing short-cut nitrification-denitrification to a two-stage PN/A process. Although no external anammox sludge inoculation was performed to save seeding sludge cost, the start-up was successfully achieved in about 9 months (273 days) based on a three-step method of “AnAOB enrichment - sludge acclimation - capacity doubling”. During start-up, the relative abundance of AnAOB (Candidatus_*Kuenenia*) increased from near zero to 12.0%. After start-up, the total inorganic nitrogen (TIN) removal load reached 0.74 kgN/(m^3^•d), with a total nitrogen removal efficiency of over 90%. Compared to the traditional nitrification-denitrification process, the PN/A process remarkably reduces the addition of organic chemicals and aeration energy consumption, saving approximately 4.2 million yuan (RMB) in operational costs annually. In summary, this research provides a full-scale reference for the start-up of the PN/A process treating sludge HSAD reject water.

## Highlights

• A 480 m^3^/d full-scale two-stage PN/A treating sludge HSAD reject water was successfully started up.
• A three-step method “AnAOB enrichment - sludge acclimation - capacity doubling” was utilized.
• The start-up was accomplished within 9 months without external anammox sludge inoculation.
• TIN removal load of 0.74 kgN/(m³•d) was achieved after start-up.

## Graphical abstract

**Figure:**
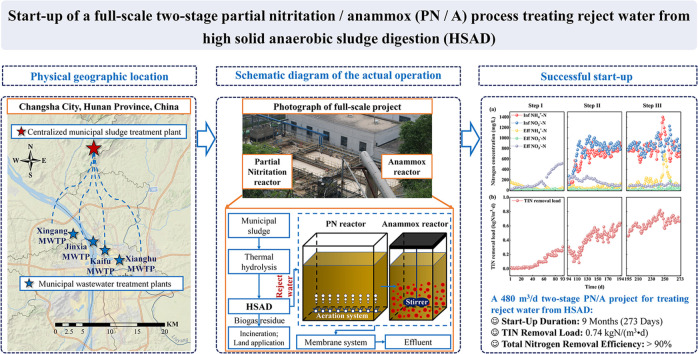

## 1 Introduction

High solid anaerobic digestion (HSAD) is an emerging and promising direction in the evolution of traditional anaerobic digestion (AD) technology. AD is recognized as an environmentally friendly method for the treatment and energy recovery from biodegradable organic wastes. The process can be categorized into two main types based on the total solids (TS) concentration of the treated solid waste: low solid anaerobic digestion (LSAD) with a TS content <15%, and high solid anaerobic digestion (HSAD) with a TS content >15% (Xu et al., 2021). In many countries, particularly those in development, the AD application for sludge from wastewater treatment plants (WWTPs) is relatively limited due to challenges such as high investment in anaerobic reactors, large quantities of reject water produced, and high operational costs (Yang et al., 2015). These issues necessitate further advancements in AD technology to fully harness its developmental and applicational potential (Fagbohungbe et al., 2015). Consequently, in recent years, HSAD has emerged as a promising solution. This technology has seen rapid development and presents several advantages, including high volumetric loading, reduced reactor investment, lower reject water production, and higher system efficiency (Duan et al., 2012; Xue et al., 2015). Moreover, the higher solids content aids in reducing the sludge volume and decreasing transportation costs. This is particularly economically beneficial for countries (e.g. China) that adopt centralized sludge treatment plants to which dewatered sludge from nearby WWTPs was transferred (Miyanoshita et al., 2009; Ghorbanpour et al., 2023).

The partial nitritation/anammox (PN/A) process is considered the most sustainable and efficient for nitrogen removal from sludge AD reject water. Compared with traditional processes such as Anoxic-Oxic (AO) which requires high chemicals and energy consumption, PN/A can theoretically save 100% of the organic source and more than 60% of the aeration, while also reducing the excess sludge production. Currently, there are over 200 full-scale PN/A process applications for sludge reject water treatment worldwide, mostly distributed in Europe and North America. However, it should be noted that these applications generally treat reject water from LSAD, instead of HSAD (Lackner et al., 2014; Cao et al., 2017).

The application of the PN/A process for the HSAD reject water faces more significant challenges. Compared to LSAD, the HSAD system demonstrated an enrichment effect due to its increased solid content (Akinbomi et al., 2022; W. Li et al., 2023). Accordingly, reject water from HSAD system, especially when combined with thermal hydrolysis pretreatment (THP), contains a higher concentration of organic matter with potentially toxic and harmful substances, which can inhibit the microbial communities in the PN/A process and lead to negative impacts such as poor mass transfer, low diffusion coefficients, and high viscosity (Xu et al., 2021). The chemical oxygen demand (COD) of HSAD reject water can be as high as 2 to 8 times that of the LSAD reject water (Barber, 2016). The high concentration of organic matter is prone to cause heterotrophic bacteria to compete with autotrophic bacteria for growth, leading to the instability of the anammox-based process (Li et al., 2020; Gong et al., 2021). Additionally, previous research indicated that the organic matter present in the reject water from sludge AD combined with thermal hydrolysis can indirectly or directly inhibit the growth of nitrite-oxidizing bacteria and anaerobic ammonium-oxidizing bacteria (AnAOB) (Zhang et al., 2018; Wang et al., 2021). Furthermore, the complex composition of HSAD reject water poses inhibitory challenges due to the presence of certain stress-inducing organics, suspended solids, and refractory organic nitrogen compounds (Figdore et al., 2011; Tang et al., 2018; Zhang et al., 2020). These challenges may significantly impair the PN/A system, suppress the activity of AnAOB, affect the system stability, and consequently easily lead to process start-up failure, which is detrimental to full-scale practical applications (Sheng et al., 2020). The start-up for full-scale application of a two-stage PN/A process treating sludge HSAD reject water has not been reported.

Previous studies have explored the start-up of the PN/A process for sludge LSAD reject water treatment, with reports such as Wang et al. (2017) investigating the start-up of PN/A treating LSAD reject water at a pilot scale, achieving a nitrogen removal load of 1.23 kgN/(m^3^·d) (Wang et al., 2017). Han et al. (2020) reported on the practical application of PN/A for the treatment of reject water after thermal hydrolysis, implementing the start-up in an integrated fixed-biofilm activated sludge reactor, with the total inorganic nitrogen (TIN) load of 0.21 kgN/(m^3^·d) and a nitrogen removal efficiency of over 85% (Han et al., 2020). Despite these researches, the successful start-up of large-scale PN/A processes still faces significant challenges due to the slow growth and environmental sensitivity of ammonia-oxidizing bacteria (AnAOB), particularly when applying PN/A to treat sludge HSAD reject water.

This study reports on the successful start-up of a full-scale 480 m^3^/d two-stage PN/A process for the treatment of sludge HSAD reject water. No external anammox sludge inoculation was performed to save seeding sludge costs. The research encompasses the following aspects: (1) presents a three-step method for starting up a two-stage PN/A project, which includes “AnAOB enrichment – sludge acclimation - capacity doubling”. This method ensures a successful start-up without the need for sludge inoculation for this large-scale project. (2) introduces the nitrogen removal performance and the material flow patterns during the start-up process. The microbial community variation was also revealed for a deeper understanding of the intrinsic mechanisms. This practical experience and the data provided are helpful for engineers and researchers aiming to deploy and optimize the performance of PN/A technology in full-scale operations for the treatment of HSAD reject water.

## 2 Results and discussion

### 2.1 A three-step method for full-scale start-up without external anammox sludge inoculation

**Fig. 1:**
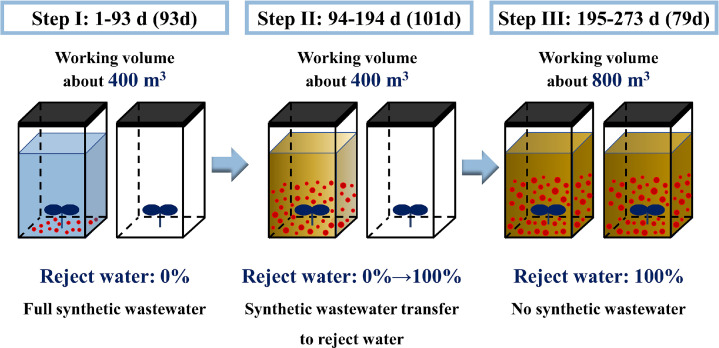
Schematic diagram of the three-step method of “AnAOB enrichment (synthetic solution) - sludge acclimation (reject water) - capacity doubling (reject water)”. **In Step I**, AnAOB was enriched by feeding synthetic solution in a single anammox reactor, with the other reactor idle, at a working volume of 400 m^3^; **In Step II**, the feeding solution gradually shifted from synthetic solution to reject water, achieving stable anammox nitrogen removal, with the working volume remaining unchanged; **In Step III**, full reject water is used and the capacity was doubled by dividing anammox sludge into two respective tanks, expanding the total working volume to 800 m^3^.

A three-step method of AnAOB enrichment – sludge acclimation - capacity doubling was used for the full-scale start-up without external anammox sludge inoculation. Given that this full-scale project is an upgrade from the existing single reactor system for high activity ammonia removal over nitrite (SHARON) process, the focus of the start-up is primarily on initiating the anammox section. Within the two-stage PN/A process, it is important to note that the influent data for the anammox reactor, effectively correspond to the effluent from the PN reactor. This correspondence implies that the influent nitrogen concentrations and other parameters are indicative of the PN reactor’s performance. The specific conditions of the three steps totaling 273 days during start-up are shown in Fig. 1.

Step I, the enrichment of the AnAOB period (lasting 93 days), involved inoculating an anammox reactor (400 m^3^) with 200 m^3^ activated sludge from the original system, resulting in a sludge concentration of 10 g/L. This step was conducted using synthetic wastewater containing sodium nitrite and ammonium chloride. The utilization of synthetic wastewater in the initial stage was pivotal, providing a stable and predictable environment that enabled the rapid cultivation and acclimation of AnAOB. This approach was preferred over the direct use of reject water, allowing for a more controlled introduction to the inherently complex and inhibitory conditions characteristic of HSAD reject water, thereby preventing any further prolongation of the system’s start-up time. Despite there being no addition of external anammox sludge, the enrichment of AnAOB was gradually achieved. Initially, the sludge demonstrated no anammox activity, but after about one month of enrichment, anammox activity emerged, and the nitrogen removal load gradually increased with the continuous AnAOB enrichment.

Step II was the sludge acclimation period for real reject water (101 days). The influent to the reactor was gradually changed from synthetic wastewater to actual reject water, acclimating the anammox sludge to the reject water. The influent was a mixture of reject water and synthetic wastewater at first, with the proportion of reject water increasing from none to all, until the influent was entirely reject water. After stabilization, the daily treatment of reject water reached 200–240 m^3^.

Step III, the capacity doubling period (79 days), was necessary due to the large scale of reject water and the presence of two anammox reactors in the project. After initiating a single anammox reactor in step II, half of the sludge was transferred to the second anammox reactor, effectively doubling the treatment capacity of reject water. After stable operation, the total working volumes of the two reactors were 1100 m^3^ and 800 m^3^, respectively.

### 2.2 The performance during the three-step start-up of two-stage PN/A treating sludge HSAD reject water

**Fig. 2:**
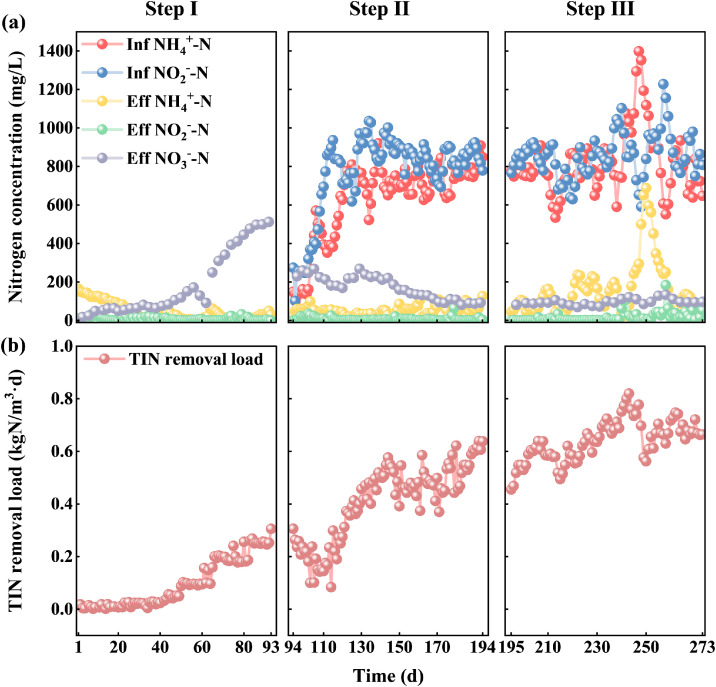
Performance during three-step start-up of two-stage PN/A. Nitrogen removal performance (a); nitrogen removal load (b). Step I: 93 days; Step II: 101 days; Step III: 79 days (273d in total).

The full-scale two-stage PN/A project is successfully started up by the three-step method, and the performance of its nitrogen concentration and TIN removal load during start-up are shown in Fig. 2a and Fig. 2b, respectively. Meanwhile, the HRT variation during start-up is shown in **Figure S1**.

Step I is the enrichment for AnAOB, lasting a total of 93 days, with both the PN reactor and the anammox reactor having a working volume of approximately 400 m³ each. As this project does not have external sludge inoculation of AnAOB, there was no anammox activity at the very beginning. Meanwhile, during this phase of step I, synthetic wastewater was used to enrich AnAOB, and to prevent the loss of bacteria, only limited effluent was discharged, which is why the number of influent and effluent concentration measurements were not applicable. However, the daily quantity of added chemicals was recorded, enabling the calculation of the TIN removal load, which reflects the operational situation. The effluent NH_4_^+^-N is around 150 mg/L, and the concentrations of NO_2_^−^-N and NO_3_^−^-N are both around 30 mg/L. At day 42, the effluent NO_2_^−^-N and NH_4_^+^-N levels dropped simultaneously to about 15 and 50 mg/L, respectively, indicating the first appearance of anammox activity. At this time, the TIN removal load was still relatively low, only around 0.03 kgN/(m³·d). Meanwhile, the effluent NO_3_^−^-N gradually increased to about 80 mg/L. By day 93, the effluent NH_4_^+^-N concentration decreased to 30 mg/L, NO_2_^−^-N concentration was below 1 mg/L, and due to the anammox reaction consuming NH_4_^+^-N and NO_2_^−^-N to produce NO_3_^−^-N, the NO_3_^−^-N concentration increased to about 500 mg/L, and the TIN removal load gradually increased to 0.31 kgN/(m³·d). It is worth noting that since this project did not inoculate with external anammox sludge due to economic considerations, step I of cultivation and acclimation of AnAOB is required. If inoculated anammox sludge was used, this step can be skipped or further shortened. Thanks to the availability of external sludge inoculation, the startup process can be further shortened, typically ranging from one to three months (Ni and Zhang, 2013). Analysis of microbial diversity in the anammox section revealed that the abundance of AnAOB increased from less than 0.1% in step I to 0.9% in step II, indicating that the AnAOB was successfully enriched.

Following step I of AnAOB enrichment, step II involved transitioning the influent from synthetic wastewater to reject water from sludge HSAD, lasting a total of 101 days. Due to the high concentrations of NH_4_^+^-N and NO_2_^−^-N in the reject water, step II gradually reduced the proportion of synthetic wastewater and increased the reject water flow to acclimate the anammox sludge, until the system operated stably. At the beginning of step II, the anammox activity experienced a decline due to the presence of organic compounds in the reject water that are inhibitory to the PN/A process. These compounds, resulting from the treatment processes of HSAD, negatively impacted microbial activity. Consequently, this led to an increase in the effluent concentrations of NH_4_^+^-N and NO_2_^−^-N, and a decrease in TIN removal load to 0.1 kgN/(m³·d). This situation also highlights the strategic advantage of initially enriching the system with synthetic wastewater to cultivate the microbial community before gradually introducing the reject water, which can minimize the negative impact and allow the microbial community to adapt to the more challenging conditions of the reject water. As the sludge acclimated, around the 20th day of step II, the AnAOB gradually adapted to the influent reject water environment, with effluent concentrations of NH_4_^+^-N and NO_2_^−^-N returning to the levels at the end of step I. In the later stage of step II, the influent NH_4_^+^-N and NO_2_^−^-N concentrations were maintained at around 720 mg/L and 850 mg/L, respectively, and the effluent NO_3_^−^-N concentration was reduced to below 100 mg/L, with the TIN removal load increasing to a maximum of 0.64 kgN/(m³·d) and an average of 0.41 kgN/(m³·d). After stable operation in step II, approximately 200–240 m³ of HSAD reject water was treated daily. It is worth noting that AnAOB are autotrophic, and the high COD concentration in the influent reject water of this project (3000–4000 mg/L) could lead to the growth of heterotrophic bacteria, competing with AnAOB and inhibiting their growth. Accordingly, an anaerobic period was added into each operational cycle in the PN stage to increase the COD removal, contributing to the stability of this two-stage PN/A system (Zhang et al., 2024). Moreover, by utilizing the capabilities of the PN stage to treat organic compounds resulting from THP and HSAD (Zhang et al., 2018), this study was able to create conditions that not only mitigated the inhibitory effects on AOB and AnAOB but also supported the growth of functionally beneficial microbes in the subsequent anammox stage. This two-stage PN/A system, therefore, not only ensures the stability of the process but also optimizes the treatment efficacy.

In step III, the working volume of the anammox section reactor was expanded to 800 m³, with a total operation of 79 days. In the later stage of step III, fluctuations in the concentrations of NH_4_^+^-N within the influent and effluent were observed. These variations are likely attributable to the elevated NH_4_^+^-N levels in the reject water influent, which exceeded 1000 mg/L. This was possibly exacerbated by the substantial sludge discharge from the PN section, leading to an increase in NH_4_^+^-N concentration in the anammox tank. Such an increase can adversely affect the activity of AnAOB, subsequently impacting NH_4_^+^-N removal efficiency. This is a common challenge in full-scale biological wastewater treatment systems. However, by making necessary adjustments to the sludge discharge, the nitrogen removal efficiency rapidly recovered within a short period, highlighting the robustness of the process and its capacity to adapt to influent variability. This resilience is a crucial characteristic for ensuring the stability and reliability of large-scale wastewater treatment operations. During the stable operation of step III, the two-stage PN/A treated about 480 m³ of sludge reject water daily, with the TIN removal load of the two-stage reactors ranging from 0.60 to 0.75 kgN/(m³·d), peaking at 0.82 kgN/(m³·d), and averaging 0.64 kgN/(m³·d), with an average nitrogen removal rate of 90.0%. After continuing to operate stably through step III and an additional two months, the average nitrogen removal load could stabilize at 0.74 kgN/(m³·d). The stable data on the long-term stable operation of the system are shown **in Figure S2**.

**Fig. 3:**
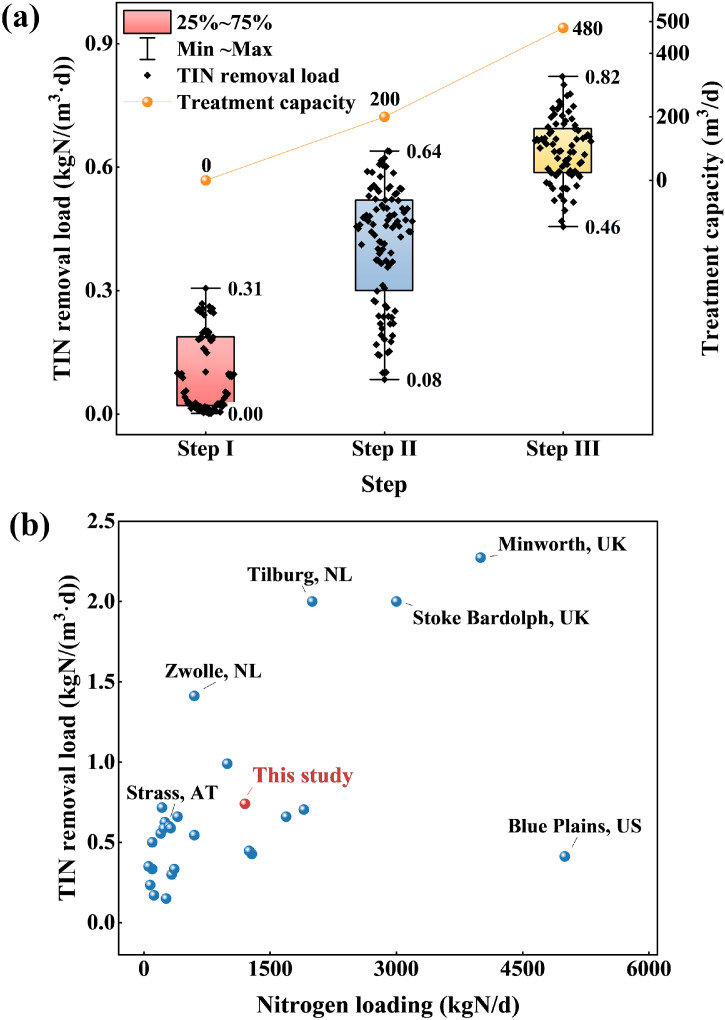
Three-step nitrogen removal load and treatment capacity for a two-stage PN/A (a) and the general situation of global sludge reject water PN/A projects (b).

In general, a two-stage PN/A process to treat 480 m³/d HSAD reject water without anammox sludge inoculation was successfully started up, as depicted in Fig. 3a. The start-up was meticulously carried out over three steps, spanning a total of 273 days. It is important to note that in the context of anammox processes, even with the knowledge and experience gained over the years since the first full-scale anammox reactor, which took 3.5 years to start without the inoculation of external anammox sludge (Abma et al., 2007; Van Der Star et al., 2007), achieving a startup within nine months represents a substantial improvement. The nitrogen removal load was incrementally elevated, reaching a peak of 0.64 kgN/(m³·d), and ultimately stabilizing at 0.74 kgN/(m³·d). The optimal operational parameters, as determined by prior laboratory-scale experiments, were stringently controlled in the full-scale start-up, with the PN tank and anammox tank maintained at temperatures of 30–36 °C and 33–36 °C, respectively. The pH levels were tightly regulated between 6.5–8.3 for the PN and 7.5–8.2 for anammox, while the dissolved oxygen (DO) concentrations were kept at 0.5–1 mg/L and close to 0 mg/L for the respective reactors.

According to the previous studies (Lackner et al., 2014; Ali and Okabe, 2015; Cao et al., 2017; Parde et al., 2024), the scale of global sludge anammox projects was usually not very large, as shown in Fig. 3b. Most achieved total nitrogen loading in range of 0–500 kgN/d categorized as small size, treating reject water from certain WWTPs, while some in range of 500–1000 kgN/d categorized as medium size. Only a few projects obtain large scale with loading over 1000 kgN/d. For this research, due to the reject water coming from a centralized sludge AD plant treating dewatered sludge from several nearby WWTPs, the PN/A scale was large and its loading after start-up reached as high as 1200 kgN/d. Besides, most full-scale PN/A applications generally used a TIN removal load below 0.8 kgN/(m^3^•d) while this project successfully achieved a high TIN removal load of 0.74 kgN/(m^3^•d), positioning it at the forefront in terms of both operational scale and nitrogen removal efficiency.

### 2.3 Microbial community analysis of the two-stage PN/A process

High-throughput sequencing was used to evaluate the microorganisms’ diversity in both the PN and anammox stages during each start-up step. Generally, the relative abundance of AnAOB (Candidatus_*Kuenenia*) kept getting more and more as the start-up process went along.

**Fig. 4:**
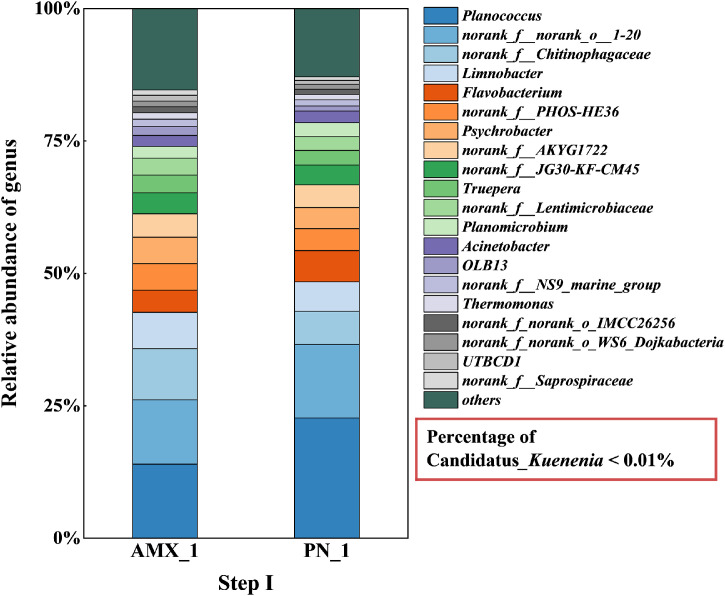
Microbial community relative abundance at the genus level in the two-stage PN/A reactor during step I.

As shown in Fig. 4, during the first start-up step, the anammox reactor was predominantly inhabited by *Planococcus*, which made up 14.0% of the microbial community. These bacteria can play a significant role in processes like denitrification and act as nitrate-reducing bacteria, which are also important for breaking down complex organic matter (Luo et al., 2021). In contrast, the abundance of the AnAOB genus Candidatus_*Kuenenia* was extremely low, less than 0.01%. This low abundance is attributed to the lack of inoculation with anammox sludge during the start-up. At this time, the system lacked anammox activity, resulting in lower nitrogen removal capacity and loading. The genera with higher abundance in the PN stage were similar to those in the anammox stage, which may be due to the fact that both stages were inoculated with sludge from the original SHARON process.

**Fig. 5:**
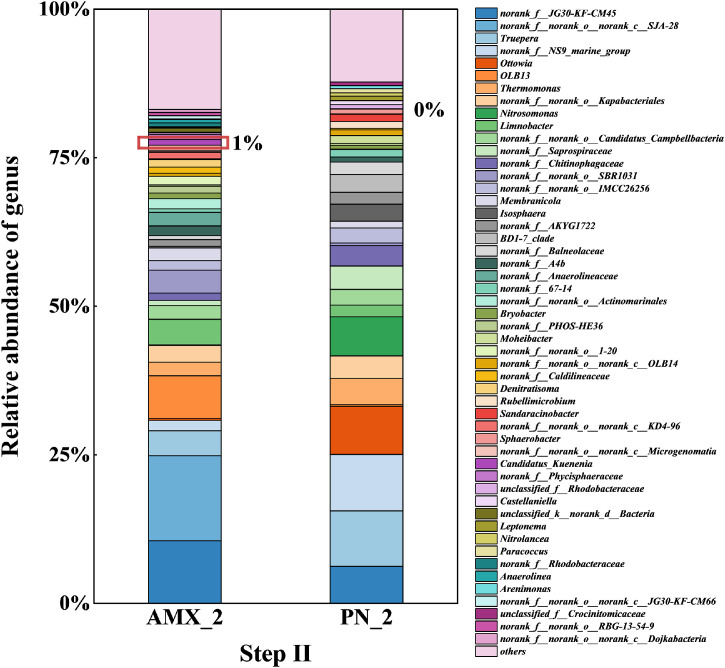
Microbial community relative abundance at the genus level in the two-stage PN/A reactor during step II.

As shown in Fig. 5, during step II of start-up, the main microbial genera in the anammox stage were norank_f_norank_o_norank_c_*SJA-28* (14.3%), norank_f_*JG30-KF-CM45* (10.6%), *OLB13* (7.2%). All three are heterotrophic denitrifying bacteria, playing key roles in promoting microbial aggregation and reducing excess nitrate in the anammox system (Xiao et al., 2022). *OLB13* also plays a crucial role in nitrite accumulation (Zhang et al., 2021), which helps maintain system stability and the stable operation of the subsequent anammox section (Su et al., 2022). Notably, in step II, the abundance of the Candidatus_*Kuenenia* genus increased to 0.9%, indicating that AnAOB continues to enrich as the influent shifts from synthetic wastewater to reject water, with the system’s nitrogen removal load averaging 0.41 kgN/(m³·d). In the PN stage, the main microbial genera were norank_f_*NS9_marine_group* (9.5%), *Truepera* (9.3%), and *Ottowia* (8.1%). Previous research revealed that norank_f_*NS9_marine_group* might be a new AOB genus with ammonia-oxidizing capabilities, occupying an advantageous position among AOB due to its strong adaptability and continuously providing a substrate for the subsequent AnAOB (Wang et al., 2020). The most important functional genus in the PN stage, *Nitrosomonas*, had an abundance of 6.6%. Additionally, *Truepera* and *Ottowia*, being denitrifying bacteria, contribute to the removal of organic compounds and nitrogen, thereby protecting the activity of the AnAOB in the subsequent anammox stage (Wang et al., 2020).

**Fig. 6:**
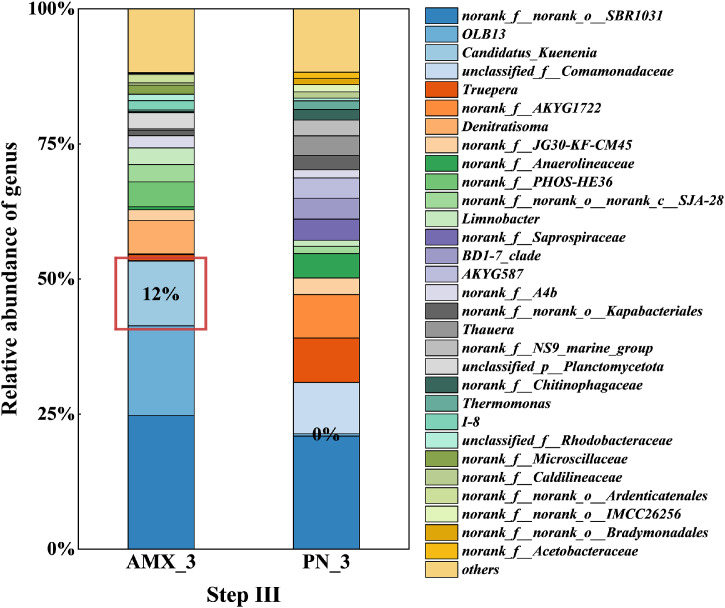
Microbial community relative abundance at the genus level in the two-stage PN/A reactor during step III.

In step III, as shown in Fig. 6, the main microbial genera in the anammox stage were norank_f_norank_*o_SBR1031* (24.7%), *OLB13* (10.6%), Candidatus_*Kuenenia* (12.0%). The abundance of Candidatus_*Kuenenia* significantly increased compared to step II after about 200 days of start-up. This indicates that AnAOB continuously enriches in the reactor, increasing in abundance and achieving the start-up of the anammox process. Norank_f_norank_*o_SBR1031*, a heterotrophic bacterium, can utilize cell debris and extracellular proteins to promote the aggregation of anammox sludge (H. Li et al., 2023). In the PN stage, the main microbial genera were norank_f_norank_*o_SBR1031* (20.9%), unclassified_f_*Comamonadaceae* (9.5%), *Truepera* (8.3%). Previous research indicated that unclassified_f_*Comamonadaceae* plays an important role in maintaining the activity of AnAOB under adverse conditions, such as low temperatures (Lv et al., 2020). In summary, at the genus level, the main functional bacteria in the anammox stage are AnAOB (Candidatus_*Kuenenia*) and some denitrifying bacteria (DNB), while in the PN stage, they are nitritation bacteria (such as *Nitrosomonas*, norank_f_*NS9_marine_group*) and DNB. The mixed system enhances the stability of the system, and the microbial community structures of the two-stage system are significantly different. The two-stage PN/A process achieves the separation of functional bacteria, improving the simplicity and efficiency of operation, and providing a strong microbial basis for the start-up and operation of the anammox process.

### 2.4 Operating costs saved by the two-stage PN/A process compared with the traditional AO process

This study investigated the cost savings after applying the PN/A process to treat sludge HASD reject water. The two-stage PN/A process has a high economic value because it significantly reduces the chemical (carbon source) consumption and sludge disposal cost of the reject water treatment process. The PN/A process was modified to have a smaller footprint and higher treatment efficiency. The two-stage process can save the consumption of additional carbon sources and aeration volume compared with the traditional AO process and SHARON process. According to the current total scale of 800 m^3^/d reject water in the system, if methanol is added as a carbon source, the calculation shows that the implementation of the PN/A process can save 11,530 yuan (RMB) per day, namely 4.21 million RMB per year (see **Section S2** of the Supplementary Material for details). In specific, the reduction in organic chemical costs accounts for 79.2% of the total savings, with the decrease in aeration contributing an additional 20.8%. Overall, the two-stage PN/A process for the treatment of high solids sludge AD reject water demonstrated obvious economic benefits.

## 3 Conclusion

This study demonstrated the successful start-up process of a 480 t/d PN/A project without anammox sludge inoculation and treating HSAD reject water from a centralized dewatered sludge treatment plant. Although no external anammox sludge inoculation, the start-up was successfully achieved in about 9 months (273 days) based on a three-step method of “AnAOB enrichment - sludge acclimation - capacity doubling”. The two-stage PN/A process demonstrated high efficiency, with a TIN removal load of 0.64 kgN/(m³·d) at the end of the start-up phase and a nitrogen removal rate exceeding 90%. As the two-stage PN/A process stabilized, the TIN removal load increased to an average of 0.74 kgN/(m³·d). During the start-up, the main functional bacteria in the anammox stage, AnAOB (Candidatus_*Kuenenia*), continuously enriched, with their abundance growing from near zero to 12.0%. Implementing the PN/A process resulted in annual savings of 4.2 million RMB. This research provides a full-scale reference for the start-up of the PN/A process treating sludge HSAD reject water.

## 4 Materials and methods

### 4.1 The centralized HSAD of sludge in wastewater treatment plants and the characteristics of its reject water quality

**Fig. 7:**
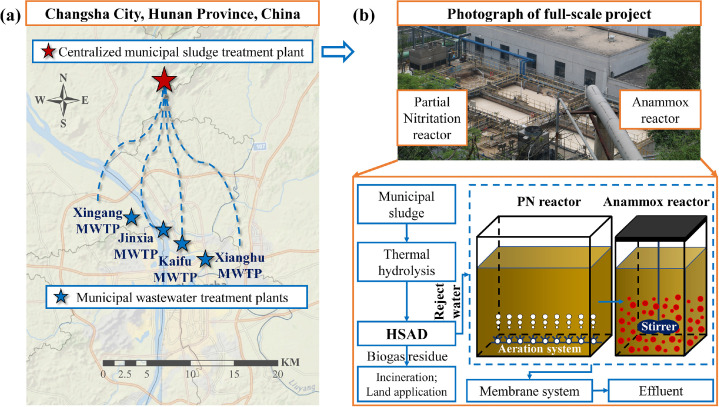
The centralized HSAD sludge treatment project. (a) Schematic diagram of the centralized sludge treatment mode; (b) Actual photo and process flow diagram of the upgraded two-stage PN/A process.

This study focuses on the reject water from a centralized HSAD project of sludge in Changsha City, Hunan Province, China. The project has a total sludge treatment capacity of 500 t/d (with a moisture content of 80%), collecting dewatered sludge from four wastewater treatment plants in the urban area of Changsha City (Fig. 7a), and employs a process of “high-temperature thermal hydrolysis + HSAD + sludge dewatering + sludge drying”. Dewatered sludge from different municipal wastewater treatment plants is transported by dedicated vehicles to this sludge project for centralized treatment. The sludge first undergoes high-temperature thermal hydrolysis pretreatment to promote the solubilization of organic matter in the sludge, which is conducive to subsequent anaerobic methanation. After thermal hydrolysis, the sludge is cooled to approximately 58 °C through a heat exchanger and then enters two separate concrete anaerobic digestion tanks with an effective volume of about 10,000 m^3^ each, with a designed retention time of about 22 days and a feed solids content higher than 10%. The biogas produced from anaerobic digestion is purified and desulfurized before being stored in a double-membrane gas holder, and then supplied to the site’s boilers, drying systems, and generators. The sludge after AD is subjected to a dewatering machine system for solid-liquid separation, with the solid part having a moisture content lower than 60%, and then further dried by belt thermal drying to a moisture content lower than 40%, after which it is used as additional soil for landfill cover.

After solid-liquid separation, the reject water enters the water treatment system. The main characteristics of the sludge AD reject water are high NH_4_^+^-N concentration, low C/N ratio, and low alkalinity-to-nitrogen ratio. The basic water quality characteristics of the sludge anaerobic digestion liquid are shown in **Table S1**. The original process of the water treatment system was a SHARON process. After denitrification, it is treated by a two-stage AO system, then enters ultrafiltration (UF) and reverse osmosis (RO), and finally meets the Class 1A standard for wastewater discharge, with the treated wastewater reused in the boiler room.

Based on the original process, this study successfully initiated a two-stage PN/A process using existing structures. Considering factors such as cost, the start-up process of the anammox reactor did not inoculate with external sludge, but instead enriched AnAOB on-site first, and then gradually started up the reactor. In contrast, the PN section was directly inoculated with sludge from the original system for start-up. It is important to highlight the presence of a long-term operational anammox pilot-scale device on-site, with an effective volume of 30 m^3^. The pilot device’s effluent is discharged into the anammox reactor structure that is being started up on-site, with the pilot effluent carrying a small amount of suspended sludge. For further details regarding the pilot scale, reference can be made to the work by Zhang et al (Zhang et al., 2024), where a comprehensive description of the related conditions and results is provided*.* In addition, by optimizing the use of existing structures and thanks to the inherent space-saving advantages of the PN/A process, the start-up process did not affect the operation of the original process, thus achieving a non-stop and non-reduction start-up process.

### 4.2 The structure of tanks used for upgrading to the two-stage PN/A

The two-stage PN/A process in this study utilized existing structures, upgrading from a SHARON process to a two-stage PN/A process, which mainly includes a partial nitritation reactor and an anammox reactor, as shown in Fig. 7b. The original aeration reactor was reconstructed into a partial nitritation reactor with dimensions of length × width × height = 13.5 m × 13.5 m × 6.0 m, with a volume of approximately 1100 m^3^. The former biological anoxic tank was transformed into the anammox section, consisting of two tanks, each with a working volume of length × width × height = 8 m × 7.6 m × 6 m, approximately 400 m^3^ each. No carriers were added. Furthermore, the hyperbolic stirrer is selected for mixing, ensuring that the environmental conditions are consistently favorable for the growth and activity of the anammox bacteria, contributing to the stability and efficiency of the start-up process.

### 4.3 Analytical methods

The concentrations of NH_4_^+^-N, NO_2_^−^-N, and NO_3_^−^-N were ascertained following filtration through 0.45 μm membranes, employing Nessler’s reagent photometric method for NH_4_^+^-N, the N-(1-naphthyl)-ethylenediamine photometric method for NO_2_^−^-N, and ultraviolet spectrophotometry for NO_3_^−^-N, respectively.

The microbial community analysis was conducted using PCR technology with primers 338F (5′-ACT CCT ACG GGA GGC AGC AG-3′) and 806R (5′-GGA CTA CHV GGG TWT CTA AT-3′) to amplify the V3-V4 region of the 16S rRNA gene. High-throughput sequencing was employed to analyze the microbial community composition. The amplicon sequencing was performed on the Illumina MiSeq platform by Majorbio BioPharm Technology Co., Ltd. (Shanghai, China). The raw sequencing reads from this study was deposited into the Sequence Read Archive (SRA) at the NCBI website under the BioProject accession number PRJNA1132401.

## CRediT authorship contribution statement

**Shuyan Zhou:** Writing – original draft, Methodology, Formal analysis, Data curation. **Hui Gong:** Writing – review & editing, Writing – original draft, Supervision, Conceptualization. **Enhui Xu:** Formal analysis, Data curation. **Xiang Chen:** Formal analysis, Data curation. **Xiankai Wang:** Formal analysis, Data curation. **Hang Wang:** Data curation. **Danyang Zhu:** Data curation. **Yanyan Zhang:** Data curation. **Jing Yang:** Data curation. **Guowei Gu:** Supervision, Methodology. **Xiaohu Dai:** Supervision, Methodology, Conceptualization.

## Declaration of competing interest

The authors declare that they have no known competing financial interests or personal relationships that could have appeared to influence the work reported in this paper.

## Data Availability

Data will be made available on request

## Appendix Supplementary materials

Image, application 1

## References

[bib0001] Abma W.R., Schultz C.E., Mulder J.W., Van Der Star W.R.L., Strous M., Tokutomi T., Van Loosdrecht M.C.M. (2007). Full-scale granular sludge Anammox process. Water Sci. Technol..

[bib0002] Akinbomi J.G., Patinvoh R.J., Taherzadeh M.J. (2022). Current challenges of high-solid anaerobic digestion and possible measures for its effective applications: a review. Biotechnol. Biofuels..

[bib0003] Ali M., Okabe S. (2015). Anammox-based technologies for nitrogen removal: advances in process start-up and remaining issues. Chemosphere.

[bib0004] Barber W.P.F. (2016). Thermal hydrolysis for sewage treatment: a critical review. Water. Res..

[bib0005] Cao Y., Van Loosdrecht M.C.M., Daigger G.T. (2017). Mainstream partial nitritation–anammox in municipal wastewater treatment: status, bottlenecks, and further studies. Appl. Microbiol. Biotechnol..

[bib0006] Duan N., Dong B., Wu B., Dai X. (2012). High-solid anaerobic digestion of sewage sludge under mesophilic conditions: Feasibility study. Bioresour. Technol..

[bib0007] Fagbohungbe M.O., Dodd I.C., Herbert B.M.J., Li H., Ricketts L., Semple K.T. (2015). High solid anaerobic digestion: operational challenges and possibilities. Environ. Technol. Innov..

[bib0008] Figdore B., Bowden G., Stinson B., Wett B., Hell M., Bailey W., Carr J., Der Minassian R., Murthy S. (2011). Treatment of dewatering sidestream from a thermal hydrolysis-mesophilic anaerobic digestion process with a single-sludge deammonification process. Proc. Water Environ. Fed..

[bib0009] Ghorbanpour S., Farzadkia M., Kermani M., Kalantary R.R., Pasalari H. (2023). Hydraulic feasibility of discharging sludge from a local WWTP to a centralized WWTP through sewage network lines. Heliyon..

[bib0010] Gong H., Ding J., Wang S., Xu E., Xue Y., Gu G. (2021). Optimizing granular anammox retention via hydrocycloning during two-stage deammonification of high-solid sludge anaerobic digester supernatant. Sci. Total Environ..

[bib0011] Han X., Zhang S., Yang S., Zhang L., Peng Y. (2020). Full-scale partial nitritation/anammox (PN/A) process for treating sludge dewatering liquor from anaerobic digestion after thermal hydrolysis. Bioresour. Technol..

[bib0012] Lackner S., Gilbert E.M., Vlaeminck S.E., Joss A., Horn H., Van Loosdrecht M.C.M. (2014). Full-scale partial nitritation/anammox experiences – an application survey. Water. Res..

[bib0013] Li H., Cai T., Gao Y., Dai Q., Liu X., Chen X., Lu X., Zhen G. (2023). Long-term performance, microbial evolution and spatial microstructural characteristics of anammox granules in an upflow blanket filter (UBF) treating high-strength nitrogen wastewater. Bioresour. Technol..

[bib0014] Li Jialin, Li Jianwei, Peng Y., Wang S., Zhang L., Yang S., Li S. (2020). Insight into the impacts of organics on anammox and their potential linking to system performance of sewage partial nitrification-anammox (PN/A): a critical review. Bioresour. Technol..

[bib0015] Li W., Gupta R., Zhang Z., Cao L., Li Y., Show P.L., Gupta V.K., Kumar S., Lin K.-Y.A., Varjani S., Connelly S., You S. (2023). A review of high-solid anaerobic digestion (HSAD): From transport phenomena to process design. Renew. Sustain. Energy Rev..

[bib0016] Luo D., Meng X., Zheng N., Li Y., Yao H., Chapman S.J. (2021). The anaerobic oxidation of methane in paddy soil by ferric iron and nitrate, and the microbial communities involved. Sci. Total Environ..

[bib0017] Lv Y., Pan J., Huo T., Li J., Liu S. (2020). Enhance the treatment of low strength wastewater at low temperature with the coexistence system of AnAOB and heterotrophic bacteria: Performance and bacterial community. Sci. Total Environ..

[bib0018] Miyanoshita T., Oda N., Hayashi N., Fujiwara M., Furumai H. (2009). Economic evaluation of combined treatment for sludge from drinking water and sewage treatment plants in Japan. J. Water Suppl.: Res. Technol.-Aqua.

[bib0019] Ni S.-Q., Zhang J. (2013). Anaerobic ammonium oxidation: from laboratory to full-scale application. Biomed. Res. Int..

[bib0020] Parde D., Behera M., Dash R.R., Bhunia P. (2024). A review on anammox processes: Strategies for enhancing bacterial growth and performance in wastewater treatment. Int. Biodeterior. Biodegradat..

[bib0021] Sheng L., Lei Z., Dzakpasu M., Li Y.-Y., Li Q., Chen R. (2020). Application of the anammox-based process for nitrogen removal from anaerobic digestion effluent: A review of treatment performance, biochemical reactions, and impact factors. J. Water. Process. Eng..

[bib0022] Su B., Liu Q., Liang H., Zhou X., Zhang Y., Liu G., Qiao Z. (2022). Simultaneous partial nitrification, anammox, and denitrification in an upflow microaerobic membrane bioreactor treating middle concentration of ammonia nitrogen wastewater with low COD/TN ratio. Chemosphere.

[bib0023] Tang Y., Li X., Dong B., Huang J., Wei Y., Dai X., Dai L. (2018). Effect of aromatic repolymerization of humic acid-like fraction on digestate phytotoxicity reduction during high-solid anaerobic digestion for stabilization treatment of sewage sludge. Water. Res..

[bib0024] Van Der Star W.R.L., Abma W.R., Blommers D., Mulder J.-W., Tokutomi T., Strous M., Picioreanu C., Van Loosdrecht M.C.M. (2007). Startup of reactors for anoxic ammonium oxidation: Experiences from the first full-scale anammox reactor in Rotterdam. Water. Res..

[bib0025] Wang G., Xu X., Zhou L., Wang C., Yang F. (2017). A pilot-scale study on the start-up of partial nitrification-anammox process for anaerobic sludge digester liquor treatment. Bioresour. Technol..

[bib0026] Wang S., Gong H., Ding J., Xu E., Cui R., Yang D., Gu G., Dai X. (2021). Unbalanced inhibition on granular and mixed anammox sludge by different molecular weight fractions of unbiodegradable proportion of sludge anaerobic digestion reject water. J. Water. Process. Eng..

[bib0027] Wang W., Xie H., Wang H., Xue H., Wang J., Zhou M., Dai X., Wang Y. (2020). Organic compounds evolution and sludge properties variation along partial nitritation and subsequent anammox processes treating reject water. Water. Res..

[bib0028] Xiao R., Zhu W., Zheng Y., Xu S., Lu H. (2022). Active assimilators of soluble microbial products produced by wastewater anammox bacteria and their roles revealed by DNA-SIP coupled to metagenomics. Environ. Int..

[bib0029] Xu Y., Gong H., Dai X. (2021). High-solid anaerobic digestion of sewage sludge: achievements and perspectives. Front. Environ. Sci. Eng..

[bib0030] Xue Y., Liu H., Chen S., Dichtl N., Dai X., Li N. (2015). Effects of thermal hydrolysis on organic matter solubilization and anaerobic digestion of high solid sludge. Chem. Eng. J..

[bib0031] Yang G., Zhang G., Wang H. (2015). Current state of sludge production, management, treatment and disposal in China. Water. Res..

[bib0032] Zhang D., Feng Y., Huang H., Khunjar W., Wang Z.-W. (2020). Recalcitrant dissolved organic nitrogen formation in thermal hydrolysis pretreatment of municipal sludge. Environ. Int..

[bib0033] Zhang L., Hao S., Wang Y., Lan S., Dou Q., Peng Y. (2021). Rapid start-up strategy of partial denitrification and microbially driven mechanism of nitrite accumulation mediated by dissolved organic matter. Bioresour. Technol..

[bib0034] Zhang Q., Vlaeminck S.E., DeBarbadillo C., Su C., Al-Omari A., Wett B., Pümpel T., Shaw A., Chandran K., Murthy S., De Clippeleir H. (2018). Supernatant organics from anaerobic digestion after thermal hydrolysis cause direct and/or diffusional activity loss for nitritation and anammox. Water. Res..

[bib0035] Zhang Y., Gong H., Zhu D., Lu D., Zhou S., Wang Y., Dai X. (2024). A two-stage partial nitritation-denitritation/anammox (PN-DN/A) process to treat high-solid anaerobic digestion (HSAD) reject water: Verification based on pilot-scale and full-scale projects. Water. Res. X..

