# Supplementary material for: Start-up of a full-scale two-stage partial nitritation/anammox (PN/A) process treating reject water from high solid anaerobic sludge digestion (HSAD)
Source: Water Res X. 2024 Sep 23;25:100259. doi: 10.1016/j.wroa.2024.100259 (PMC11490804; doi:10.1016/j.wroa.2024.100259)
Supplement: Supplementary file 1 [file mmc1.docx]

Supporting Information

Start-up of a full-scale two-stage partial nitritation/ anammox (PN/A) process treating reject water from high solid anaerobic sludge digestion (HSAD)

**Shuyan Zhou^a^, Hui Gong^a *^, Enhui Xu^a^, Xiang Chen^b,c^, Xiankai Wang^b,c^, Hang Wang^b,c^, Danyang Zhu^a^, Yanyan Zhang^a^, Jing Yang^a^, Guowei Gu^a^, Xiaohu Dai^a^**

^a^ College of Environmental Science and Engineering, State Key Laboratory of Pollution Control and Resources Reuse, Tongji University, Shanghai 200092, China

^b^ YANGTZE Eco-Environment Engineering Research Center, China Three Gorges Corporation, Wuhan 430010, China

^c^ National Engineering Research Center of Eco-environment Protection for Yangtze River Economic Belt, Wuhan 430010, China

* Corresponding author: 1239 Siping Road, Shanghai 200092, P.R China

Email address: [gonghui001@tongji.edu.cn](mailto:gonghui001@tongji.edu.cn)

## S1 The performance during the three-step start-up of two-stage PN/A treating sludge HSAD reject water

| 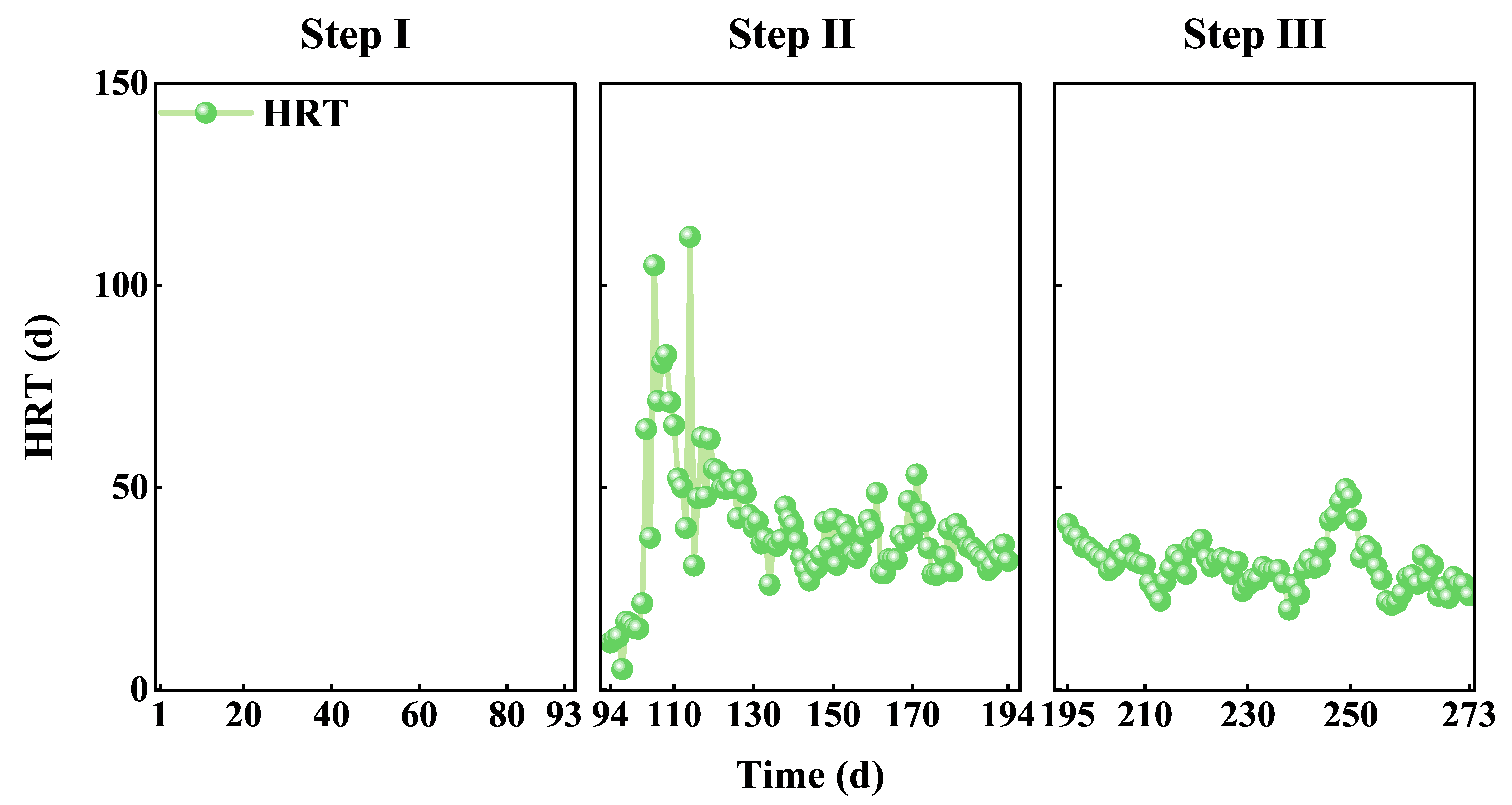 |
| --- |
| **Figure S1**. HRT variation during the start-up of full-scale two-stage PN/A. |

The HRT variation during the three-step start-up of two-stage PN/A treating sludge HSAD reject water is shown in **Figure S1**.

| (a) | 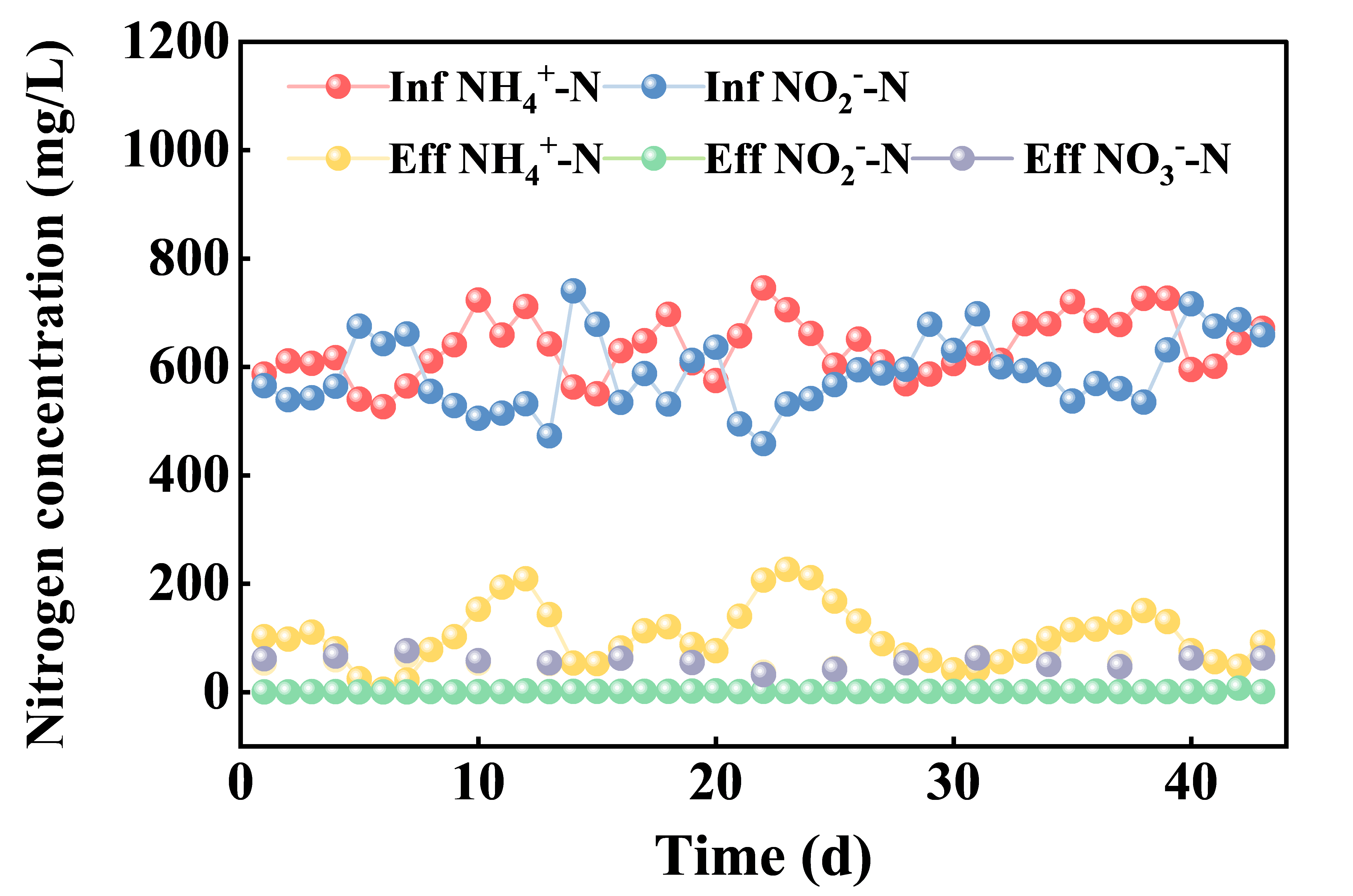 |
| --- | --- |
| (b) | 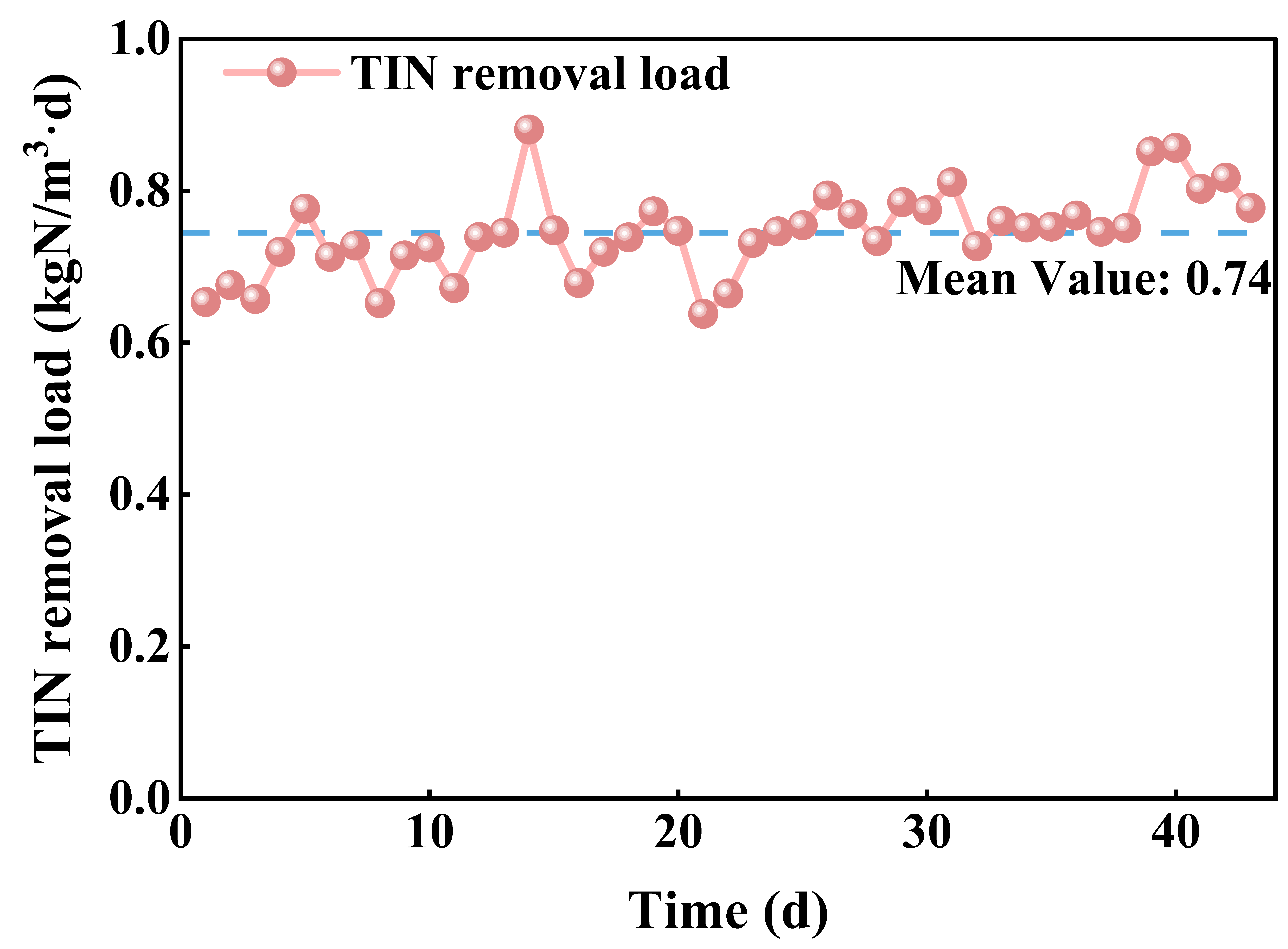 |
| **Figure S2**. Performance long-term stable operation of two-stage PN/A. Nitrogen removal performance (a); TIN removal load (b). | |

## S2 The detailed economic analysis of the two stages PN/A process

Compared to the traditional nitrification-denitrification process, the two-stage PN/A process can save on the addition of external carbon sources and aeration energy consumption. The reaction processes of the traditional nitrification-denitrification and the PN/A process are illustrated in **Figure S3**.

| 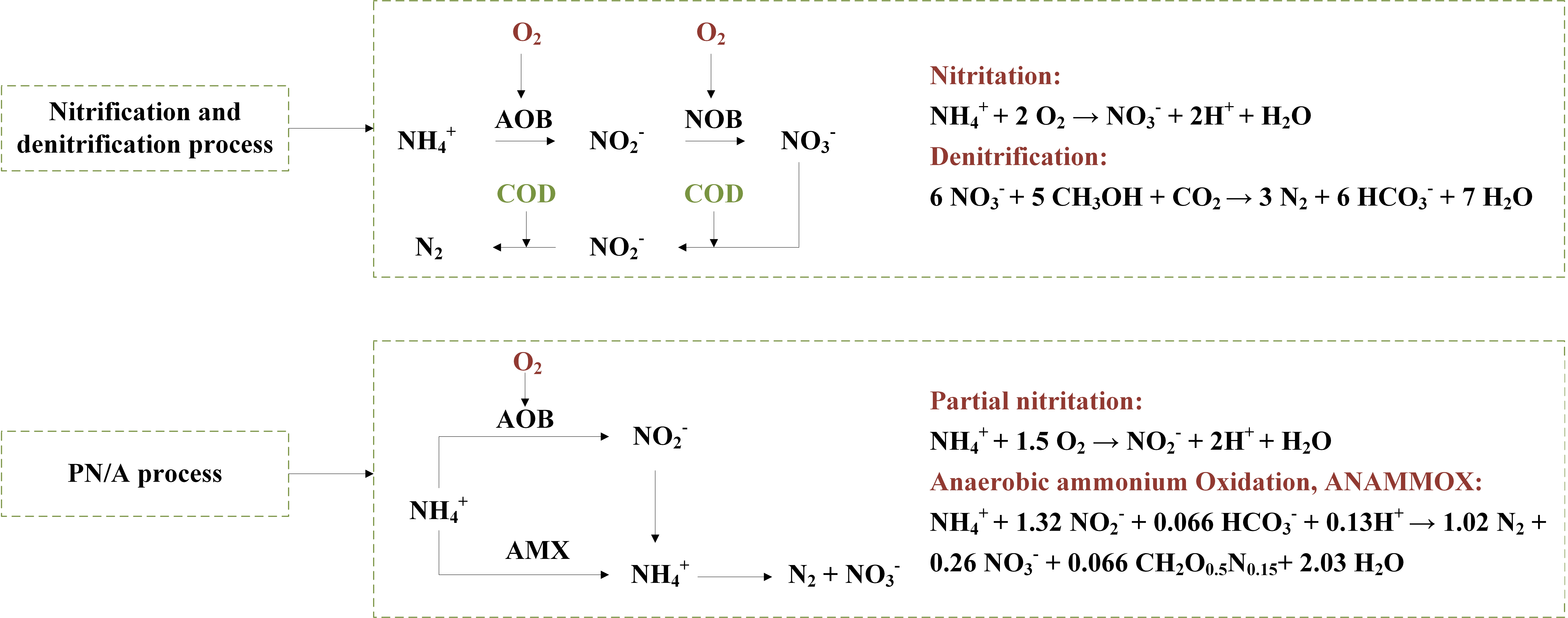 |
| --- |
| **Figure S3**. Schematic comparison of the traditional nitrification-denitrification and PN/A processes. |

This study on the two-stage PN/A process takes into account the costs of carbon source addition and aeration power consumption. The operational costs of each process and the savings achieved by the two-stage PN/A process are calculated using the following equations (1) to (8):

| $E_{cost}=\Delta E_{cost}\times E_{O_{2}}\times E_{consumption}$ | (1) |
| --- | --- |
| $S_{cost}=\Delta S_{cost}\times S_{CH3OH}$ | (2) |
| $L=Q\times({{NH}_{4}^{+}}_{Influent}-{{NH}_{4}^{+}}_{effluent})$ | (4) |
| $E_{O_{2}, typical}=4.57\times L$ | (5) |
| $E_{O_{2}, PN/A}=3.43\times L\times\frac{62}{18}$ | (6) |
| $S_{CH3OH, typical}=2.47\times L$ | (7) |
| $M_{save}=M_{typical}-M_{PN/A}=(E_{cost, typical}+S_{cost, typical})-(E_{cost, PN/A})$ | (8) |

**Where:**

*E_cost_: Total aeration cost, RMB/d.*

*△E_cost_ : Cost per kWh electricity consumption, RMB/kWh.*

*E_O2_: Oxygen demand, kg/d.*

*E_O2, typical_: Oxygen demand for nitrification-denitrification, with a conversion factor of 4.57, i.e., 1 kg NH_4_^+^-N requires 4.57 kg O_2_ to be oxidized to NO_3_^-^-N, kg/d.*

*E_O2, PN/A_: Oxygen demand for nitrification-denitrification, with a conversion factor of 3.43, i.e., 1 kg NH_4_^+^-N requires 3.43 kg O_2_ to be oxidized to NO_2_^-^-N, kg/d. It is assumed that 55% of NH_4_^+^-N is converted to NO_2_^-^-N in the PN/A process.*

*E_consumption_: Electrical consumption per kg of oxygen, kWh/kg.*

*S_cost_ : Total cost of external carbon source methanol, RMB/d.*

*△S_cost_: Cost per kg of external carbon source methanol, RMB/kg.*

*S_CH3OH_ : Amount of external carbon source methanol added, kg/d.*

*S_CH3OH, typical_: Cost of carbon source methanol for nitrification-denitrification, with a conversion factor of 2.47, i.e., 1 g NO_3_^-^-N requires 2.47 g CH_3_OH for reduction to N_2_, RMB/kg.*

*L : Influent NH_4_^+^-N in the reject water, kg/d.*

*Q : Daily reject water treatment volume, m^3^/d.*

*NH_4_^+^-N_Influent_: Influent ammonia nitrogen concentration, mg/L.*

*NH_4_^+^-N_effluent_: Effluent ammonia nitrogen concentration, mg/L.*

*M_save_: Total cost savings, RMB/d.*

*M_typical_: Cost of traditional nitrification-denitrification, RMB/d.*

*M_PN/A_: Cost of two-stage PN/A, RMB/d.*

*S_cost,typical_: Total cost of external carbon source methanol for traditional nitrification-denitrification, RMB/d.*

*E_cost,typical_: Total aeration cost for traditional nitrification-denitrification, RMB/d.*

*E_cost,PN/A_: Total aeration cost for PN/A, RMB/d.*

The daily reject water treatment volume is calculated based on a full load of 800 m^3^/d, with an average influent ammonia nitrogen concentration of 820 mg/L and an average effluent ammonia nitrogen concentration of 150 mg/L. Theoretical calculations using stoichiometric formulas and equations (1) to (8) indicate that for traditional nitrification-denitrification nitrogen removal, the total daily oxygen demand is 2,450 kg/d. Assuming an oxygen utilization rate of 15% and an electrical consumption of 0.5 kWh/kg O_2_, the total electricity consumption is 8,165 kWh/d. With methanol as the external carbon source, the total amount added is 4,566 kg/d. In the two-stage PN/A process, when 55% of NH_4_^+^-N is converted to NO_2_^-^-N in the partial nitrification step, the total oxygen demand is 1,011 kg/d, and the total electricity consumption is 3,371 kWh/d. With the cost of each kg of external carbon source methanol at 2 RMB/kg and the cost of each kWh of electricity at 0.5 RMB/kWh, according to equation (8):

$$M_{save}=M_{typical}-M_{PN/A}=(E_{cost, typical}+S_{cost, typical})-(E_{cost,PN/A})=11530 RMB/d$$

Consequently, the two-stage PN/A process can achieve daily savings of approximately 11,530 RMB, which equates to an annual saving of about 4.21 million RMB after implementing the PN/A process for the treatment of reject water from HSAD of sludge. It is important to note that these figures are theoretical calculations. During the start-up phase, the actual consumption of carbon sources and electricity is higher. Moreover, in actual operation, the denitrification reaction may not strictly adhere to the theoretical stoichiometric ratios, so the specific costs could be slightly higher than the theoretical values. However, in general, the PN/A process offers significant economic benefits and substantially reduces operational costs.

## S3 Characterization of reject water from centralized HSAD of sludge

**Table S1.** Characterization of reject water from HSAD of sludge

| **Parameter** | **Value** | **Unit** |
| --- | --- | --- |
| NH_4_^+^-N | 2000-3500 | mg/L |
| COD | 3000-4000 | mg/L |
| pH | 8.0 | - |
| SS | 400 | mg/L |
| Alkalinity | 3300 | mg/L |
| EC | 20.00 | ms/cm |
